# Editorial: Global excellence in gastroenterology: Africa

**DOI:** 10.3389/fmed.2022.1121276

**Published:** 2023-01-09

**Authors:** Reda Elwakil, Ponsiano Ocama, Violet Kayamba, Yasser Fouad, Olusegun Ojo

**Affiliations:** ^1^Tropical Medicine Department, Ain Shams University, Cairo, Egypt; ^2^Internal Medicine Department, Makerere University, Kampala, Uganda; ^3^Internal Medicine Department, Zambia University, Lusaka, Zambia; ^4^Endemic Medicine Department, Minia University, Minia, Egypt; ^5^Department of Morbid Anatomy, Obafemi Awolowo University, Ife, Nigeria

**Keywords:** Africa, global excellence, gastroenterology research, gastroenterology practice, Frontiers in Medicine topics

Africa is the world's second-largest and second-most populous continent, after Asia. At about 30.3 million km^2^ (11.7 million square miles) including adjacent islands, it covers 6% of Earth's total surface area and 20% of its land area (1). With 1.4 billion people (2, 3) as of 2021, it harbors about 18% of the world's human population. Africa's population is the youngest all over the world (4).

In the past, measurements of African health were not always accurate or representative. The African continent was home to a particularly diverse and deadly set of tropical diseases. Even in 2020, 96 percent of malaria deaths globally occurred within the African continent, with most fatalities occurring before the age of five in children (5).

Recently, the situation improved as the third United Nations Sustainable Development Goal 2030 (UN SDG 3) highlighted support of research and development of vaccines and medicines for communicable and non-communicable diseases that primarily affect developing countries, as it provide access to affordable essential medicines and vaccines, in accordance with the Doha Declaration on the TRIPS and Public Health, which affirms the right of developing countries to use the full provisions in the Agreement on Trade-Related Aspects of Intellectual Property Rights (TRIPS) regarding flexibilities to protect public health, and, in particular, provide access to medicines for all (6).

The work on the UNSDG 3 in many African countries was reflected on many positive outputs in the African health sector which has included the recent introduction of an effective vaccine against childhood malaria, the rapid recovery of life expectancies due to AIDS drugs and substantial increases in health expenditure across the continent since 2000 (5).

On September 30th 2021, Frontiers in Medicine launched a special topic under the title “*Global excellence in gastroenterology practice: Africa*” to highlight recent progress achieved in Gastroenterology specialty in Africa. The topic received 11 manuscript submissions. Four manuscripts were rejected: Two manuscripts were rejected due to being non-related to the topic subject and the other two failed to have the endorsement of two reviewers to be published according to the standards of Frontiers in Medicine. The seven manuscripts accepted for publication include five research articles, one opinion article and one mini review article.

In an original research on a cohort of Zambian children suffering from stunting non-responsive to nutritional support, Mulenga et al. studied the small intestinal epithelial changes occurring in such disease. They performed confocal laser endomicroscopy (CLE) in 75 children and collected intestinal biopsies for histology in 91 children. Morphometry was carried out on well-orientated mucosa and 3 biopsies were examined on electron microscopy. CLE demonstrated substantial leakage from circulation to gut lumen in 73 (97%) children. Histology consistently showed characteristic changes of environmental enteropathy: villus blunting, lamina propria and epithelial inflammation, and depletion of secretory cells. The use of the new imaging modality CLE and examining intestinal biopsies by morphometry and electron microscopy helped to reveal the secrets of children with stunting non-responsive to nutritional support which is a common disease in all developing countries. For all developing countries, it is estimated that 32 and 20% of children (<5 years old) are under-height (height-for-age *Z* score < −2) and under-weight (weight-for-age *Z* score < −2) for age (7).

On the other hand, another research by Schillinger et al. reported a new breath test using 13C-sucrose for the non-invasive assessment of environmental enteropathy in Zambian adults with promising results.

The study included in the topic conducted by Dabbous et al. at Ain Shams University, Cairo, Egypt addressed the risk factors and the management of biliary stones post living donor liver transplant and its effect on graft outcome. Biliary complications were previously reported in 39.0% post living donor liver transplantation (8). Dabbous et al. identified risk factors in both donors and recipients and proved that endoscopic management is the best option with the best results in those cases. They also proved that with precise and prompt management they could avoid morbidity and have similar survival rate as the control group.

The reported prevalence of PHG varies from 3.7 to 75% in patients with portal hypertension and from 15.1 to 100% in patients with liver cirrhosis (9).

In the article of Alarfaj et al. included in the current topic, the authors studied *H. pylori* infection in 80 Egyptian cirrhotic patients with portal hypertensive gastropathy (PHG) and 80 cirrhotic patients without PHG (controls) at Mansoura University Hospital, Mansoura, Egypt. They reported that the prevalence of *H. pylori* infection was significantly higher in patients with PHG (*P* < 0.001). The severity of PHG was associated with *H. pylori* infection (*P* < 0.001). They found that the response to eradication therapy of *H. pylori* infection was better in patients without PHG (*P* = 0.045).

In the current topic, a basic research article from Egypt conducted by El Sobky et al. documented the role of lnc RNA in causing *de novo* lipogenesis (DNL) and fat droplet accumulation in hepatocytes as a cause of non-alcoholic fatty liver disease (NAFLD). Their study showed for the first time the impact of miR-6155p and H19 on the mTOR/SREBP1c axis and its functional impact on lipid droplets and triglyceride accumulation in hepatocytes. These findings might pave the way for using ncRNAs as potential therapeutic targets in the management of fatty liver.

In conclusion there is a great potential for high standard gastroenterology practice and research in Africa, as shown in the previously presented articles from the topic, provided that all unmet needs are addressed. The unmet needs include: Increasing funds to health care sectors, increasing research budgets in all African countries whether through the support of native governments or through international funding agencies support in an equitable manner, prioritization of research projects according to local needs, improvement of the documentation and establishing registries for malignant and other diseases, increasing collaborative research at local, regional, continental and international levels and lastly the Africans should aim high to publish high standard research in highly citable medical journals to keep in pace with the international standards.

## Author contributions

RE wrote the draft. PO, VK, YF, and OO reviewed the manuscript. All authors contributed to the article and approved the submitted version.
